# Immortelle Essential-Oil-Enriched Hydrogel for Diabetic Wound Repair: Development, Characterization, and In Vivo Efficacy Assessment

**DOI:** 10.3390/pharmaceutics16101309

**Published:** 2024-10-08

**Authors:** Marijana Andjic, Jovana Bradic, Aleksandar Kocovic, Marko Simic, Veljko Krstonosic, Ivan Capo, Vladimir Jakovljevic, Nevena Lazarevic

**Affiliations:** 1 Department of Pharmacy, Faculty of Medical Sciences, University of Kragujevac, 34000 Kragujevac, Serbia; andjicmarijana10@gmail.com (M.A.); simic.marko.kg@gmail.com (M.S.); nevenasdraginic@gmail.com (N.L.); 2 Center of Excellence for Redox Balance Research in Cardiovascular and Metabolic Disorders, 34000 Kragujevac, Serbia; drvladakgbg@yahoo.com; 3 Department of Pharmacy, Faculty of Medicine, University of Novi Sad, 21000 Novi Sad, Serbia; veljkokrst@yahoo.co.uk; 4 Department of Histology and Embryology, Faculty of Medicine, University of Novi Sad, 21000 Novi Sad, Serbia; ivan.capo@mf.uns.ac.rs; 5 Center for Medical and Pharmaceutical Investigations and Quality Control, University of Novi Sad, 21000 Novi Sad, Serbia; 6 Department of Physiology, Faculty of Medical Sciences, University of Kragujevac, 34000 Kragujevac, Serbia; 7 Department of Human Pathology, 1st Moscow State Medical, University IM Sechenov, 119991 Moscow, Russia

**Keywords:** *Helichrysum italicum*, essential oil, hydrogel, wound healing, oxidative stress, histopathology

## Abstract

**Background:** Alarming data revealed that 19% to 34% of adults with diabetes mellitus develop chronic wounds, which are characterized by impaired healing and a higher risk of infections. Inspired by the traditional use of immortelle for wound healing and the lack of scientific evidence regarding how it thoroughly influences tissue regeneration, we aimed to formulate a hydrogel loaded with immortelle essential oil and assess its effectiveness on diabetic excision wounds. **Methods:** The rheological properties of the hydrogel, an in vivo safety test, as well as wound healing capacity, were determined in rats with induced diabetes and excision wounds. Diabetic rats were divided into four groups: untreated, treated with 1% silver sulfadiazine ointment, treated with a gel base, and treated with the immortelle essential oil-based hydrogel. **Results:** It was revealed that the hydrogel exerts pseudoplastic behavior and has no potential to act as an irritant, thus highlighting its suitability for skin application. Moreover, analysis of macroscopic, biochemical, and histopathological data revealed that the immortelle essential oil-based hydrogel significantly improves wound repair. Superior re-epithelialization, scar maturation, and increased collagen fiber density were achieved after immortelle essential oil-based gel application. **Conclusions:** These findings suggest that the immortelle essential oil-based hydrogel could be a natural, safe, and effective wound-healing dressing.

## 1. Introduction

The *Helichrysum* (*Asteraceae*) genus includes over 600 species spread throughout the world, and the most well-known species from this genus are *Helichrysum italicum*, *Helichrysum stoechas*, and *Helichrysum arenarium*. *Helichrysum italicum* and *Helichrysum stoechas* can be found throughout the Mediterranean, especially in the region of the Adriatic [1,2]. *Helichrysum italicum* is an aromatic shrub that has characteristic yellow flowers and possesses a characteristic, strong smell that is reminiscent of curry. This plant is also known by names such as “perpétuas-das-areias”, “perpétuasde-Itália”, “immortelle” or “everlasting” [3]. The plant is widely used in traditional Mediterranean medicine due to its various beneficial effects in the treatment of some inflammatory and allergy conditions, hematoma, sunburn, and wound healing [4]. Extracts and essential oils made from this plant contain various chemical compounds, such as phenolic acids, acetophenones, tremetones, terpenes, α-pyrones, and flavonoids [4]. The various terpenes found in essential oils are one of their most characteristic constituents that possess antimicrobial, antioxidant and anti-inflammatory effects, which can contribute greatly to the wound healing process [5].

Wound healing represents a complex process that occurs in all organs and tissues, and the duration of healing depends on the type of injury, mediators involved, underlying disease, and local factors [6]. Wound repair involves four phases such as hemostasis, inflammation, proliferation, and remodeling which are disturbed in diabetes and significantly delay and complicate wound healing [7]. Alarming data suggest that between 19% and 34% of adults with diabetes mellitus will develop chronic wounds, such as foot ulcers. These chronic diabetic wounds are marked by delayed healing and increased risk of infections, frequently leading to lower extremity amputations. This not only affects patients’ quality of life but also significantly increases healthcare costs [8]. Current therapy involves the use of antibiotics and wound care products that, while effective in many ways, also have significant limitations, involving long healing time, high frequency of application, and occurrence of allergies or resistance after long-term use [9]. Our previous research revealed that semi-solid formulations containing immortelle essential oil demonstrated a notable effect on wound repair in the incision rat wound model that reflects its use in the healing of sutured wounds and scar formation [10]. However, there is currently a lack of evidence on how immortelle essential oil, when incorporated into hydrophilic polymers that provide a moist healing environment and controlled release of active substances, impacts unsutured excisional wounds. These wounds closely mimic acute clinical wounds and involve different healing mechanisms compared to incisional wounds [11].

In order to address the aforementioned drawbacks of diabetic wound products, the aim of this study was to prepare and characterize a natural gel loaded with immortelle essential oil and provide comprehensive insight into its wound-healing effect by assaying the relevant histological and biological parameters in diabetic rats with induced excision wounds.

## 2. Materials and Methods

### 2.1. Materials

The preparation of the gel was achieved with carbomer, propylene glycol, triethanolamine, and distilled water, which were purchased from Unichempharm, Cacak, Serbia. Standard buffer solutions for measuring pH were purchased at Mettler Toledo, Columbus, OH, USA. Ketamine, xylazine, and streptozotocin were provided by Merck, Darmstadt, Germany. Reagents for the determination of hydroxyproline content (HCl, chloramine T, Ehrlich reagent) and reagents for systemic redox state parameters (sulfanilic acid, N-(1-naphthyl)-ethylenediamine dihydrochloride, ammonium chloride, borax, 85% orthophosphoric acid, sodium nitrite, 2-thiobarbituric acid, 28% trichloroacetic acid, sodium hydroxide, tris(hydroxymethyl)aminomethane, 37% hydrochloric acid, nitro-tetrazolium blue chloride, gelatin, potassium hydrogen phosphate dehydrate, potassium dihydrogen phosphate dehydrate, sodium chloride, hydrogen peroxide, D(+)-glucose monohydrate, phenol red, horseradish peroxidase, distilled water, ethanol, disodium phosphate, sodium carbonate, ethylenediaminetetraacetic acid, epinephrine, 5,5′-dithio-bis (2-nitrobenzoic acid), ethylenediaminetetraacetic acid, metaphosphoric acid, glutathione) were purchased from Sigma-Aldrich, Darmstadt, Germany. Substances for histological analysis such as formalin, isopropyl alcohol, paraffin, and hematoxylin–eosin solution were provided by Sigma-Aldrich, Darmstadt, Germany.

### 2.2. Gel Formulation Based on Immortelle Essential Oil

Hydrophilic carbomer gel was used to prepare the gel with immortelle essential oil, with the following composition: carbomer 1 g, propylene glycol 10 g, triethanolamine as needed, and water for 100 g of the preparation. First, the carbomer was continuously mixed with water until a dispersion was formed. The resulting dispersion was further mixed with propylene glycol, after which the triethanolamine solution was added. The gel formed in this way was used as a base for the production of the gel (base gel). Furthermore, to prepare an immortelle essential oil-based gel, 100% pure immortelle essential oil, purchased from “Alekpharm”, Belgrade, Serbia, was added into the hydrophilic carbomer gel at a concentration of 0.5% [10].

### 2.3. Characterization of Gel

Measurement of the pH value was performed by direct immersion of the glass electrode of the pH-meter HI 9321 (Hanna Instruments Inc, Temse, Belgium) in the test sample at room temperature. Before starting the measurement, the apparatus was calibrated using standard buffers of pH 4, 7, and 10. Measurement of the pH value was carried out in triplicate initially, 3 days after the sample was made and after 6 months of storage at room temperature.

In order to characterize the rheology of the developed formulation, continuous and oscillatory rheological measurements were carried out. A HAAKE MARS rheometer (Thermo Scientific, Karlsruhe, Germany) was used, which was connected to a thermostat to maintain temperature. A plate/plate measuring system was used for measurement. This device has a diameter of 35 mm and a 1.000 mm distance between plates. A constant temperature of 25 ± 0.1 was kept during measurements. Continuous rheological measurements were carried out using a controlled shear rate. The gel formulation samples were initially exposed to a shear rate that was increased from 0.001 to 50 s^−1^ for 120 s. After that, the shear rate was kept constant at 60 s^−1^ for the next 60 s. The last step involved determining the behavior of the samples at a reduced shear rate from 50 to 0 s^−1^ for 120 s.

Oscillatory measurements were used to examine the viscoelastic behavior of gel formulations. Determination of the linear viscoelastic region was conducted using amplitude sweep measurements. The relation between shear stress of 0.01 to 100 Pa and elastic (G′) and viscous (G″) moduli was recorded at a constant frequency of 1 Hz. Amplitude oscillatory measurements were also used for frequency tests. Measurements were made for frequencies from 0.1 to 10 Hz at a constant shear stress of 1 Pa. These rheological observations were made immediately after the sample was made, then 3 days after that, and after 6 months. The sample was kept in storage at room temperature during that period.

In order to implement the swelling index, one gram of hydrogel was soaked into 5 mL phosphate buffer pH 5.5 in the Petri dish and left in a dry place for one hour and three hours before measuring the weight. The content was weighed at three time points and the degree of swelling was established from a formula [12]. The same process was repeated after 180 days. The index was calculated according to the following formula:

Swelling Index (SW)% = [Wt − Wo/Wo ] × 100

(SW) % = Equilibrium percent swelling

Wt = Weight of swollen gel after time t

Wo = Initial weight of gel

### 2.4. Acute Dermal Irritation

The safety of the newly formed gel was assessed using the acute dermal irritation test. This test was performed on an animal model. The animals used were healthy male *Wistar albino* rats that had a weight between 200 and 250 g. The researchers followed the guidelines of the Organization for Economic Cooperation and Development (OECD) 404 for this test. The experiment had two test groups:

1. Rats treated with 0.5% immortelle oil gel (IG)
2. Rats treated with the gel base (GB)

Both preparations were applied topically, with 500 mg applied to shaved areas of the rats’ skin. The rats were observed for any signs of irritation, edema, pruritus, and erythema, as well as tremors, salivation, convulsions, and diarrhea (that could be the signs of toxicity of the preparations). Observations were particularly focused during the first 4 h and then conducted once per day for 14 days [13,14].

### 2.5. Wound Healing Assessment

#### 2.5.1. Ethics Statement

The experimental protocol for this study was approved by the Ethics Committee of the Faculty of Medical Sciences, University of Kragujevac, Serbia (approval number: 01–6292). All experiments in this study were carried out in accordance with Good Laboratory Practice and compliance with the European Directive for the Protection of Vertebrate Animals used for experimental and other scientific purposes (86/609/EES). The experiments were carried out in the Laboratory for Pharmaceutical Technology and the Center for Experimental and Preclinical Investigation at the Faculty of Medical Sciences, University of Kragujevac, Serbia.

#### 2.5.2. Animals

A total of 32 healthy adult male *Wistar albino* rats, weighing between 250 and 300 g, were used for the in vivo wound healing experimental study. These rats were obtained from the Military Medical Academy in Belgrade, Serbia. They were housed in clean cages, maintained on a 12 h light/dark cycle in a climate-controlled room set at a temperature of 22 ± 2 °C. The animals had access to food and drinking water ad libitum.

#### 2.5.3. Induction of Diabetes and Excision Wounds

A single intraperitoneal injection of streptozotocin was used to induce diabetes in the rats. The administered dose was 50 mg/kg. The injection was prepared by dissolving streptozotocin in 1 mL of freshly prepared citrate buffer solution. The citrate buffer solution had a concentration of 0.05 M and a pH value of 4.5 [15]. The rats were starved overnight before the injection. An Accu-Check^®^ portable glucometer (Basel, Switzerland) was used to measure fasting glucose levels three days after the injection and 12 h after starvation. Animals were considered diabetic if their fasting glucose levels were above 11.1 mmol/L. Only those animals were included in the experimental protocol.

Excision wounds were made a week after diabetes was confirmed in the rats. Firstly, a mixture of xylazine (10 mg/kg) and ketamine (5 mg/kg) was administered intraperitoneally to anesthetize the animals. After that, the backs of the animals were shaved and cleaned with 70% ethyl alcohol. Lastly, a scalpel and scissors were used to create open excision wounds with the dimensions of 2 × 2 cm and 2 mm in depth [16,17]. After the excision wounds were created, the rats were housed in individual cages.

#### 2.5.4. Treatment Protocol

After the excision wounds were induced, the rats were randomly divided into four groups (eight rats in each group). Group I served as the negative control with untreated wounds. Group II served as the positive control, with wounds treated using a standard ointment containing 1% silver sulfadiazine. Groups III and IV were treated with the gel base and 0.5% immortelle essential oil-based gel, respectively.

All preparations were applied topically once daily for 21 days using sterile cotton in an amount of 0.5 g. On the 21st day, the rats were anesthetized with an intraperitoneal injection of a mixture of ketamine (10 mg/kg) and xylazine (5 mg/kg) and were then sacrificed by decapitation. In order to assay the systemic redox status of the animals, blood samples were collected during decapitation. These samples were used to obtain plasma and erythrocyte lysate samples, which were then separated and stored at −20 °C. Wound tissue samples were collected as well using a scalpel and scissors. A part of these samples was used for the determination of the hydroxyproline content, while the other part was used for histopathological analysis.

### 2.6. Estimated Parameters

#### 2.6.1. Estimation of Wound Contraction

This assay was performed by taking a photo of every rat after wound formation and then on the 7th, 14th, and 21st day after wound creation. Graph paper and ImageJ software v 1.54 were then used to measure the calculated wound area in order to ascertain the percentage of wound contraction. The following formula was used to determine the percentage of wound contraction on the aforementioned days [18]:

% Wound contraction = [(Initial wound area − Specific day wound area)/Initial wound area] × 100.

#### 2.6.2. Hydroxyproline Content Estimation

Obtained wound tissue samples were first washed with a cold saline solution and dried for 12 to 18 h at temperatures of 60–70 °C. The samples were then hydrolyzed by using 6 N HCl. Hydrolysis was performed using 1 mL HCl per 10 mg of dry tissue for 4 h at a temperature of 105 °C. The obtained hydrolyzed tissue samples were then mixed with chloramine-T and left to react for about 20 min. After this process was completed, Ehrlich reagent was added to the mixture in the amount of 1 mL. Finally, a Shimadzu UV-1800 spectrophotometer was used to spectrophotometrically determine the absorbance at 558 nm. To calculate the obtained results, a calibration curve of standard hydroxyproline expressed in mg/g of dry granulation tissue was used. The hydroxyproline content was expressed as μg/mg of dry tissue weight [19].

#### 2.6.3. Markers of Inflammation

After sacrifice, samples of the wound tissue were taken and stored at −80 °C to estimate the markers of inflammation. A sample of skin tissue (100 mg) was homogenized with 0.5 mL of cell lysis buffer. The obtained homogenates were centrifuged at 5000 rpm for 10 min at a temperature of 4 °C. The obtained supernatants were stored at a temperature of −70 °C. Determination of the concentration of inflammation markers TNF-α, IL-6, and IL-10 in the supernatant obtained from skin tissue was carried out via ELISA kit according to the instructions.

#### 2.6.4. Systemic Redox State Estimation

Determination of parameters of systemic redox state was performed using the blood samples. These samples were obtained from the jugular vein during sacrifice. The samples were centrifuged to separate plasma and erythrocytes. Plasma samples were used to determine some of the pro-oxidative markers. The determined markers were the following: superoxide anion radical, hydrogen peroxide, nitrites, and index of lipid peroxidation expressed as thiobarbituric acid reactive substances (TBARS). The antioxidative defense system component levels were assayed in the erythrocyte samples. These included the following: antioxidant enzymes like superoxide dismutase and catalase and the non-enzymatic antioxidant reduced glutathione [20].

#### 2.6.5. Wound Tissue Histology

Another set of tissue samples was used for histological analysis. Firstly, the samples were fixed in 10% buffered formalin and were stored for 24 h at 4 °C. Next, the samples underwent dehydration. For this purpose, isopropyl alcohol solutions with varying concentrations of 70%, 80%, 96%, and 100% were used. The dehydrated samples were then embedded in paraffin under vacuum. A rotating microtome (Leica, Germany) was used to then section the samples at a thickness of 5 μm.

Staining of the obtained sections was performed using hematoxylin–eosin (H&E), Masson’s trichrome (MTC), and immunohistochemical markers like rabbit anti-Iba1 with a dilution of 1:8000 (Abcam; Cambridge, UK), rabbit anti-CD34 with a dilution of 1:3500 (Abcam; Cambridge, UK), rabbit anti-MMP9 with a dilution of 1:25 (Lab Vision; Thermo Scientific, Rockford, IL, USA) and rabbit anti-collagen I with a dilution of 1:300 (Abcam; Cambridge, UK). Visualization was achieved using the Mouse and Rabbit Specific HRP/DAB (ABC) Detection IHC kit (Abcam; Cambridge, UK). For slides stained with anti-collagen I and anti-MMP9, a retrieval reaction was performed using TRIS base buffer (pH 8.2), while for anti-CD34 and anti-Iba1, citrate buffer (pH 6.0) was used. All antibodies were applied for 60 min at room temperature. Counterstaining was conducted with Mayer’s hematoxylin. Finally, slides were mounted with DPX medium (Sigma-Aldrich, Taufkirchen, Germany) and coverslipped. Histological slides were analyzed using a Leica DMLB 100 T professional biological microscope (Leica, Germany) and scanned on a digital microscope VisionTek^®^ (Sakura, Japan).

Collagen quantification in histological skin samples of the examined groups was performed using the methodology described by Chen et al. [21]. Briefly, tissue samples stained with Masson’s trichrome staining technique were analyzed using ImageJ software, version FIJI (REF). The size of all images is standardized in terms of edge length and resolution. Using the “Color Deconvolution” function, versions of each of the analyzed images (red, blue, and green) were obtained [22]. The green version of the image was used to measure the collagen surface area of each image.

### 2.7. Statistical Analysis

IBM SPSS Statistics 20.0 Desktop for Windows was utilized for statistical analysis. All data are presented as mean ± standard deviation. The Shapiro–Wilk test was employed to evaluate the distribution of the data. For normally distributed data, a one-way analysis of variance (ANOVA) was conducted, followed by Tukey’s post hoc test for multiple comparisons. In cases where the distribution deviated from normality, the Kruskal–Wallis test was employed for between-group comparisons.

## 3. Results

### 3.1. Gel Characterization

The results of pH indicate that there were no significant changes in the pH value in the exanimated period, considering that the initial pH value, measured three days after making the preparation itself, was 6.57, while the value after 6 months was 6.14.

**Figure 1:**
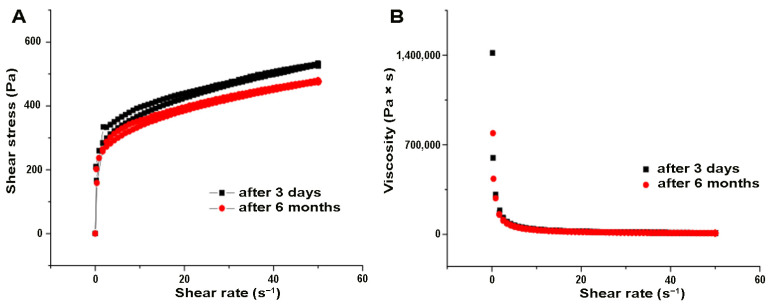
Dependence of (**A**) shear stress; (**B**) viscosity versus shear rate at different time points.

The rheological characterization of the gel with incorporated immortelle oil is presented by rheograms, which show the dependence of shear stress, i.e., viscosity, on the shear rate of the given formulation at 3 days and 6 months after production, as shown in Figure 1A,B, respectively. The presence of a hysteresis area in the shear stress versus shear rate plot confirms that the immortelle gel exhibits time-dependent, thixotropic flow. Thixotropic flow is common for gels, as bonds are formed between polymer chains that break under shear. No changes in the rheological behavior of the tested preparation after six months of storage were observed.

**Figure 2:**
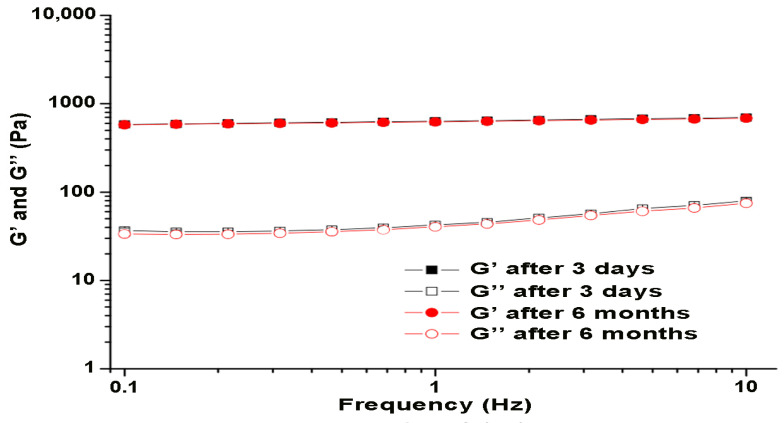
Dependence of modulus of elasticity (G′) and modulus of viscosity (G″) on frequency at different time points.

The results of the rheological measurements of elasticity (G′) and viscosity (G″) as a function of frequency are shown in Figure 2. For the given formulation, the modulus of elasticity (G′) dominated over the modulus of viscosity (G″) for the frequency range of 0.1–10 Hz. Based on this result, it can be concluded that the gel formulation with incorporated immortelle oil behaves as a viscoelastic substance within the determined frequency range. Such behavior is typical for gels due to the structure of the polymer, as well as the bonds formed between the polymer chains.

The swelling index of immortelle hydrogel on the third day and after 6 months of storage at room temperature is presented in Table 1. The immortelle hydrogel formulation showed a higher swelling index in the 3rd hour of measurement in comparison to the zero and the 1st hours immediately after the preparation of the sample.

### 3.2. Acute Dermal Irritation

The results of the test show that the developed formulation is safe to use. This can be concluded on the basis of the edema and erythema formation score, the total value of which was 0. No mortality was recorded, nor were signs of toxicity such as shivering, diarrhea, and convulsions. No signs of dermal irritation, such as itching, erythema, inflammation, and edema, were observed during the observation period of 14 days.

### 3.3. Wound Contraction

**Figure 3:**
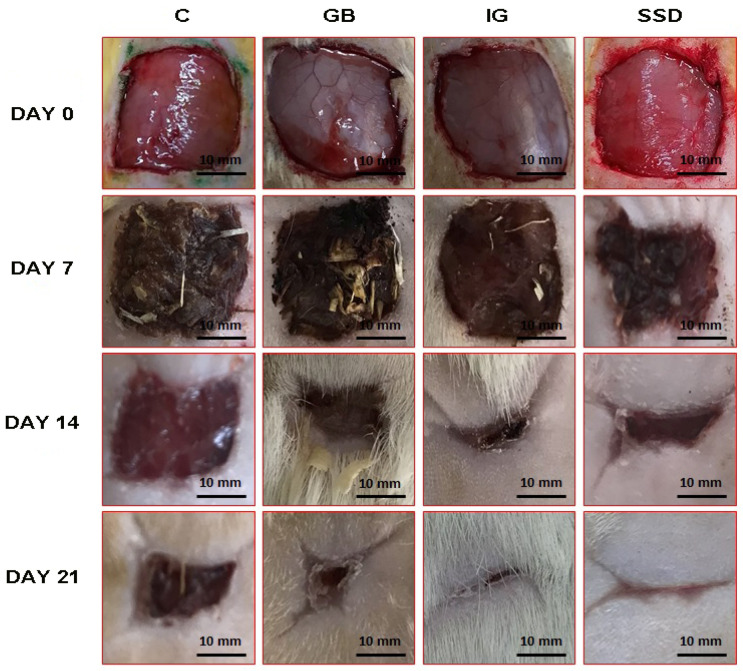
The effect of applied formulations on the healing of wounds caused by the excision method on different days (0, 7, 14, 21). C—control group; SSD—silver sulfadiazine; GB—gel base; IG—immortelle gel.

**Figure 4:**
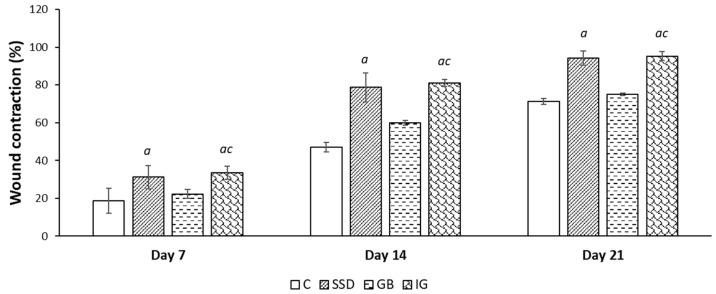
Effects of the applied formulations on wound contraction caused by the excision method. Values are expressed as mean ± standard deviation (n = 8). *^a^* Statistically significant difference at the *p* < 0.05 level compared to the C group; *^c^* statistically significant difference at the *p* < 0.05 level compared to the GB group; C—control group; SSD—silver sulfadiazine; GB—gel base; IG—immortelle gel.

The effect of the applied formulations, expressed through different moments, is shown in photographs of wounds, which indicate macroscopic changes in wound healing at different times (Figure 3) and also graphically as a percentage of wound contraction (Figure 4). Application of the gel formulation with immortelle essential oil showed faster healing of the wound compared to the negative control group and compared to the group treated with the base at all follow-up times, i.e., on the 7th, 14th, and 21st days. The significant effectiveness of the immortelle-based gel is associated with faster wound healing from the beginning to the end of the three-week application of the given formulation. There was no statistically significant difference in the surface area of the wound expressed over time between the group of animals treated with the formulation gel based on immortelle compared to the standard silver sulfadiazine.

### 3.4. Hydroxyproline Content

**Figure 5:**
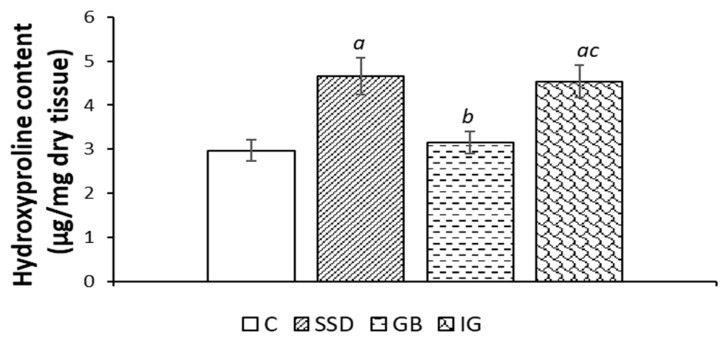
Effects of the applied formulations on the content of hydroxyproline in the wound tissue from the area of the wound caused by the excision method. Values are expressed as mean ± standard deviation (n = 8). *^a^* Statistically significant difference at the *p* < 0.05 level compared to the C group; *^b^* statistically significant difference at the *p* < 0.05 level compared to the SSD group; *^c^* statistically significant difference at the *p* < 0.05 level compared to the GB group; C—control group; SSD—silver sulfadiazine; GB—gel base; IG—immortelle gel.

A three-week application of immortelle-based gel led to a statistically significant increase in hydroxyproline content compared to the negative control group and the group of animals treated with the gel base (Figure 5).

### 3.5. Markers of Inflammation

**Figure 6:**
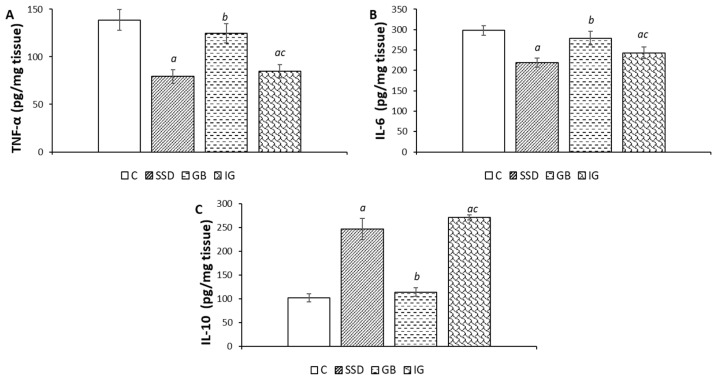
Effects of the applied formulations on the markers of inflammation in the wound tissue from the area of the wound caused by the excision method. Values are expressed as mean ± standard deviation (n = 8). *^a^* Statistically significant difference at the *p* < 0.05 level compared to the C group; *^b^* statistically significant difference at the *p* < 0.05 level compared to the SSD group; *^c^* statistically significant difference at the *p* < 0.05 level compared to the GB group; C—control group; SSD—silver sulfadiazine; GB—gel base; IG—immortelle gel.

The concentrations of pro-inflammatory markers TNF-α and IL-6, as well as anti-inflammatory IL-10, in wound tissue homogenate are shown in Figure 6. The highest concentration of the pro-inflammatory mediator TNF-α was detected in the control group. Topical administration of immortelle gel caused a significant reduction in the concentration of this parameter compared to the control group and gel base group.

At the same time, the three-week application of immortelle essential oil-based gel significantly reduced the concentration of IL-6 compared to the control and gel base groups. On the other hand, the concentration of IL-6 was almost identical compared to the SSD group.

Additionally, the statistically highest concentration of the anti-inflammatory marker, IL-10, was observed in the group of animals treated with the immortelle gel.

### 3.6. Systemic Redox Status

**Figure 7:**
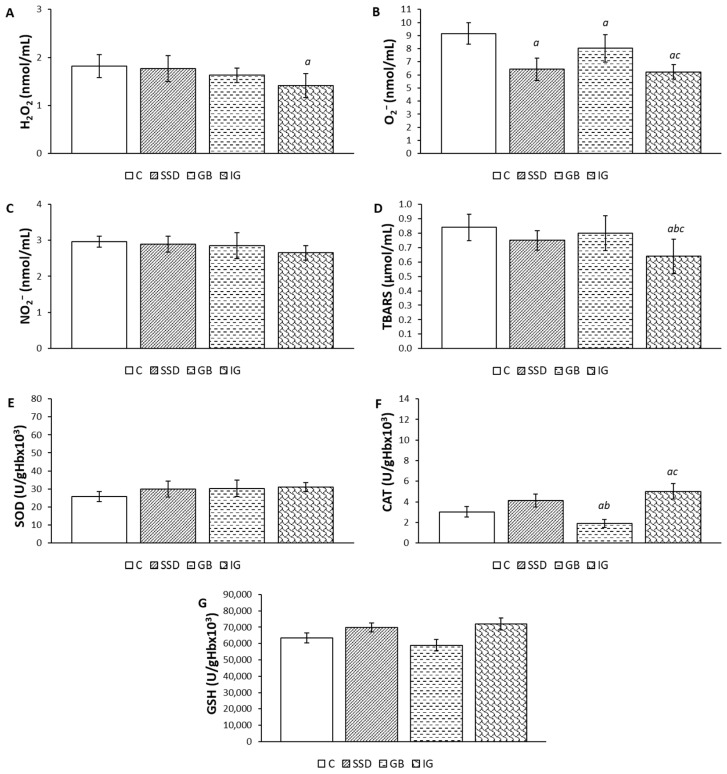
The effects of the applied formulations on pro-oxidative markers in the plasma of animals with a wound caused by the excision method: (**A**) H_2_O_2_; (**B**) O_2_^−^; (**C**) NO_2_^−^; (**D**) TBARS; (**E**) SOD; (**F**) CAT; (**G**) GSH. Values are expressed as mean ± standard deviation (n = 8). *^a^* Statistically significant difference at the *p* < 0.05 level compared to the C group; *^b^* statistically significant difference at the *p* < 0.05 level compared to the SSD group; *^c^* statistically significant difference at the *p* < 0.05 level compared to the GB group; C—control group; SSD—silver sulfadiazine; GB—gel base; IG—immortelle gel.

The highest concentration of the pro-oxidation marker O_2_^−^ was recorded in the negative control group. The application of immortelle-based gel significantly reduced the concentration of the given marker compared to the negative control and GB groups. The level of TBARS is statistically the lowest in the group of animals treated with gel with incorporated essential oil. Concerning the parameters of antioxidant protection, it is observed that CAT activity is the highest in the IG group. The given activity is significantly higher compared to the negative control and the GB group, while there is no significant difference compared to the positive control, SSD group. GSH and SOD values did not change significantly between groups (Figure 7).

### 3.7. Histological Analyses

**Figure 8:**
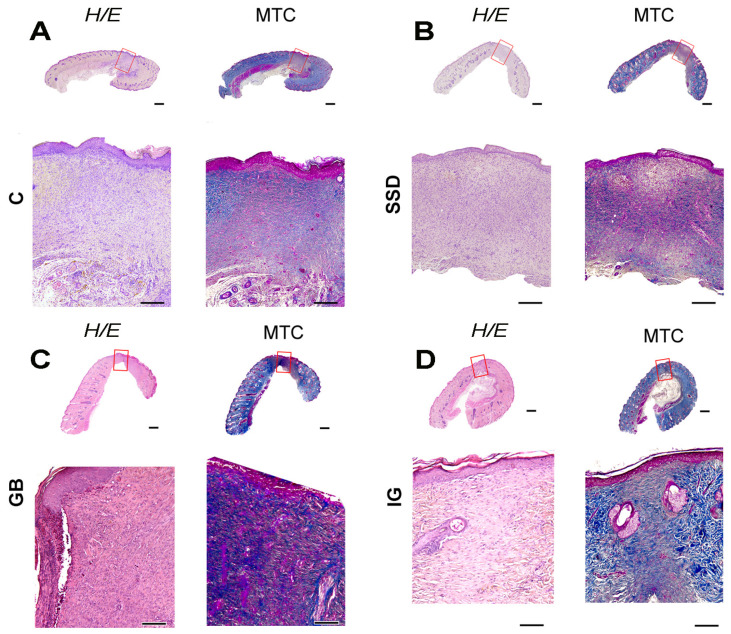
Representative view of wound tissue stained with hematoxylin–eosin (H/E) and *Masson trichrome* (MTC) technique; scale bar 1 mm. (**A**) C—control group; (**B**) SSD—silver sulfadiazine; (**C**) GB—gel base; (**D**) IG—immortelle gel.

Complete re-epithelialization of the epidermal layers was observed in groups of animals treated with gel formulations, either in the form of a substrate or based on immortelle essential oil. More noticeable changes were noted at the level of the dermis. The three-week application of gel based on essential oil contributed to the evident maturation of scars with an increased density of collagen fibers (Figure 8D). In the GB group, the scar was composed of hypercellular fibrous tissue with predominant fibroblasts (Figure 8B—H/E) and a small amount of extracellular fibrous fibers (Figure 8B—MTC). Additionally, evident scar maturation with increased collagen fiber density was observed in the immortelle gel group (Figure 8D).

**Figure 9:**
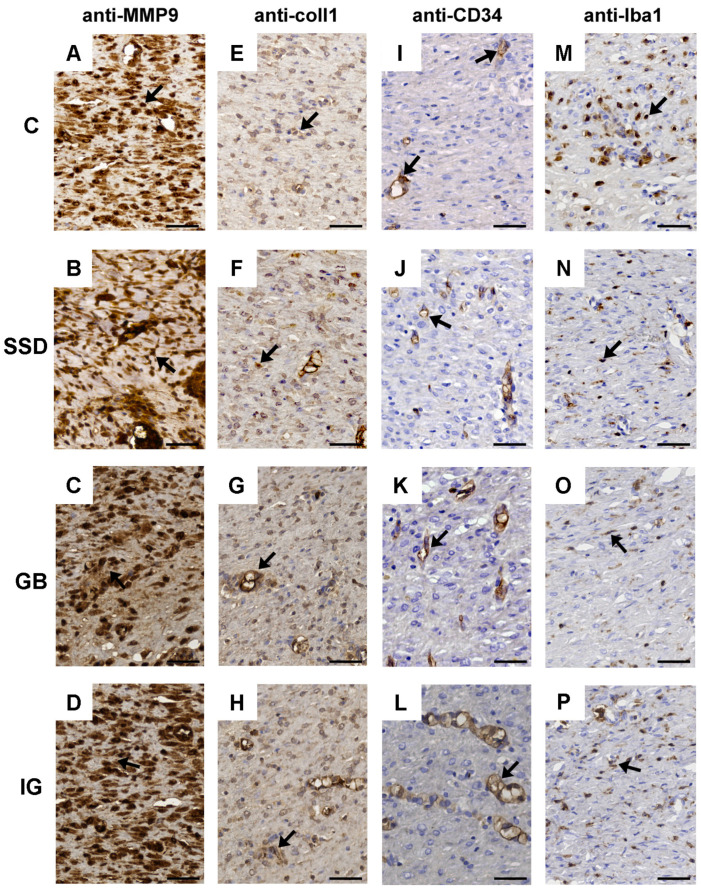
Representative immunohistochemical staining of MMP-9 (fibroblast—black arrow), (**A**–**D**), collagen I (fibroblast—black arrow), (**E**–**H**), CD34 (blood weasels—black arrow), (**I**–**L**), and Iba1 (macrophages—black arrow), (**M**–**P**) in the C group, SSD group, GB group, and IG group. Scale bar 50 μm; C—control group; SSD—silver sulfadiazine; GB—gel base; IG—immortelle gel.

Immunohistochemical staining of MMP-9, collagen 1, and CD34 in the GB group had the same characteristics in the negative and positive control groups. On the other hand, the presence of macrophages in GB was reduced compared to the negative control group. The expression of MMP-9 was intensively reduced in the group of animals treated with the gel base. The presence of collagen 1-positive fibrocytes was increased in the group treated with a preparation based on immortelle essential oil, which is in correlation with the surface of collagen (Table 2). The density of blood vessels in the GB group was still the same as in the negative control group, while in the group treated with the immortelle-based gel formulation, a prominent absence of CD34-positive structures was observed, which correlates with scar maturation. Also, the number of Iba 1-positive macrophages detected in the substrate gel group was intensively reduced in the immortelle gel group (Figure 9).

## 4. Discussion

One of the most serious long-term complications of diabetes is impaired wound healing, and diabetic patients can develop chronic diabetic ulcers, which are considered a significant health problem and social burden globally. Diabetic foot ulcers remain the primary non-traumatic reason for the loss of lower limbs and also pose a significant financial burden, where it is estimated that they take up one-third of the expenses in the treatment of diabetes [23].

Various therapeutic protocols are available for wound healing, including antibiotics, antiseptics, and anti-inflammatory agents. The significant increase in the use of medicinal plants is the result of greater safety and affordability compared to synthetic drugs [24]. Immortelle essential oil is incorporated into a large number of skin care products because it possesses antimicrobial, antioxidant, and anti-inflammatory properties and is traditionally used to treat bruises, local inflammations, edemas, and hematomas [25]. Nevertheless, apart from the selection of potent natural components such as immortelle essential oil, the wound healing efficacy of the product depends on the vehicle used and optimization of the drug delivery system. Hydrophilic polymers such as carbomer contribute to better wound management by optimizing moisture balance, protecting the wound, and supporting the healing process while allowing for the controlled delivery of terpenes present in immortelle essential oil, such as γ-curcumene, neryl acetate, α-pinene, β-selinene (11.27%), and α-selinene. [10,26]. Considering the aforementioned issues, we formulated a hydrogel wound dressing using immortelle essential oil and carbomer 940 and comprehensively examined its safety and wound healing potential in diabetic excisional wounds.

In the first part, rheological characterization of the formulated hydrogel was performed in order to predict the behavior and stability of the preparation during production, storage, and application. Determining the pH value is an indispensable parameter, both for monitoring the stability of dermocosmetic preparations and for examining the convenience, that is, the tolerability of application on the skin. The optimal pH value of formulations for dermal application should be between 5 and 6 units. The absence of a change in the pH value of the tested gel formulation during the entire storage period indicates the stability of the tested formulation. Importantly, the pH value of the immortelle oil-based gel was maintained in the 5–6 range during the six-month follow-up, indicating the biocompatibility of the formulation for use on the skin, with minimal risk of irritation or bacterial or fungal infection. Considering the percentage of immortelle essential oil in the formulation, it can be concluded that the ability to spread is mainly influenced by the base used to prepare the gel. A decrease in viscosity was observed with an increase in shear rate (Figure 1), thus revealing pseudoplastic behavior as a desirable property for gels as semisolids. At high shear rates, the gel will flow easily, thus facilitating local administration. On the other hand, at low shear rates, when the gel spreads at the application site, the preparation will have a higher consistency, restoring its original rheological properties before application [27].

In the deformation tests shown in Figure 2, large differences were observed between the elastic G′ and the loss modulus G′′, where G′ is much higher than G′′, which could indicate more prevalent viscoelastic properties of the prepared gel [28]. The frequency dependence of the modulus of elasticity and modulus of viscosity is also known as the mechanical spectrum, which is useful for determining the strength of the resulting gels. In the case of weak gels, there is a frequency dependence, and the difference between the modules is not pronounced. On the other hand, in the case of “real gels”, the G′ modulus is greater than G″ during the entire time and is therefore independent of the frequency, and exactly such features are present in the examined formulation of the gel with incorporated immortelle essential oil [29].

In order to test the safety profile of hydrogel for dermal use, the acute dermal irritation test was conducted in an animal model. The absence of erythema and edema confirms that the gel with incorporated immortelle essential oil is safe for cutaneous application and does not cause irritation. The acute dermal irritation study in animals was the crucial step in ensuring the safety of the hydrogel and encouraged the continuation of efficacy testing in excisional wounds.

The comprehensive wound-healing potential of the hydrogel loaded with immortelle was assessed through macroscopic observations, histological analysis, and biochemical testing. The results of our research showed that the percentage of wound contraction caused by the excision method was significantly higher in groups of animals treated with immortelle essential oil-based gel. The percentage of wound contraction was determined based on the calculated wound area, which was monitored at several different time points. The impressive effect of the gel with incorporated essential oil was already noted at the first time point after seven days of application. This remarkable effect of the immortelle essential oil-based gel was recorded until the last day of the experimental period, and the given results indicate a shorter re-epithelialization time in groups treated with essential oil compared to other groups (Figure 3 and Figure 4). The effectiveness of this hydrogel in the process of accelerated wound healing can be attributed to the chemical composition of the essential oil and its active compounds, such as α-pinene, β-selinene, and γ-curcumin, which play a key role in the wound healing process. It is believed that the protective effects of essential oil in wound healing are based on its anti-inflammatory, antioxidant, and antimicrobial action, as well as on the increase in collagen synthesis [30]. The wound healing potential may be attributed to the presence of terpenes, compounds with proven astringent and antimicrobial potential, which can help improve wound contraction and facilitate epithelization [31].

Collagen plays a significant role in maintaining the structural integrity of tissues, and its production and deposition are essential in certain situations, such as tissue re-epithelialization and wound healing. Hydroxyproline content determination has been used as the gold standard for quantification of collagen content because free hydroxyproline is released by the breakdown of collagen [32]. In our study, hydroxyproline content was evaluated in the tissue isolated from the previously formed wound area in order to confirm the effects of immortelle essential oil on wound healing. The results show that the highest level of hydroxyproline could be found in the wound tissue of rats treated with the immortelle essential oil-based hydrogel (Figure 5). Therefore, immortelle essential oil can significantly influence the healing process and can also provide strength to the regenerated tissue, thereby playing a vital role in tissue epithelization and homeostasis.

The inflammation phase plays a crucial role in wound healing, as it is essential for effective repair. Pro-inflammatory cytokines such as IL-6 and TNF-α are critical in regulating cell differentiation and proliferation, and they coordinate the processes of granulation tissue formation, angiogenesis, re-epithelialization, and collagen remodeling (249). Additionally, these cytokines promote leukocyte migration and proliferation within the wound, aiding in the clearance of necrotic tissue and the phagocytosis of antigens (250). IL-10, another key interleukin involved in wound inflammation, serves as an anti-inflammatory mediator by inhibiting the production of pro-inflammatory cytokines and supporting angiogenesis [33]. However, an imbalance in these cytokines can lead to chronic inflammation, which disrupts and delays the healing process [34]. Cytokine quantification from tissue samples of the wound area revealed that the anti-inflammatory effect of the immortelle essential oil-based topical formulation is associated with decreased levels of IL-6 and TNF-α. Moreover, treatment with the immortelle-based preparation increased IL-10 activity, which in turn reduces pro-inflammatory cytokine levels and scar formation. Consequently, the immortelle gel exhibited anti-inflammatory effects comparable to those of the standard treatment, silver sulfadiazine. Analysis of inflammation markers in tissue samples collected at the end of the experimental period indicates that immortelle essential oil effectively provided anti-inflammatory activity during the remodeling phase. Our findings align with existing research that highlights the anti-inflammatory properties of immortelle essential oil. Previous studies have demonstrated that the inflammatory phase restores homeostasis, while the proliferative phase involves the infiltration of fibroblasts and other connective tissue cells into the wound. These cells secrete cytokines, attract keratinocytes, and facilitate re-epithelialization [35]. Targeting and suppressing prolonged inflammation by reducing inflammatory cytokine production is a key goal for wound healing treatments, as excessive inflammation can lead to chronic wounds and increased scarring [36]. The ability of the immortelle essential oil-based gel to modulate cytokine production underscores its anti-inflammatory activity and supports its effectiveness, as demonstrated by earlier research on the essential oil and its primary components.

The histopathological analysis also showed more efficient tissue regeneration in groups of animals treated with the hydrogel gel based on immortelle essential oil. Evident scar maturation after excision, with an increase in the density of collagen fibers and an increased presence of collagen 1-positive fibrocytes, was observed in the groups treated with immortelle-based gel (Figure 9). Furthermore, immortelle essential oil-loaded hydrogel has demonstrated significant antiproliferative activity. Its wound-healing effects are aided by inhibiting collagen I and III, proteins associated with tissue remodeling. The results obtained in this research align with a previously published study where an aqueous decoction, produced after the distillation of immortelle, was effective in re-establishing tissue continuity [37]. These findings imply that the application of this formulation promotes wound healing through the stimulation of collagen deposition.

The process of angiogenesis has a significant impact on wound healing, while diabetes can cause defects in angiogenesis and collagen metabolism, which is presumed to play an important role in delayed wound healing [38]. The pronounced reduction in the number of Iba-positive macrophages and the distinct absence of CD 34-positive structures reflects the substantial progress in wound healing in animals treated with the essential oil-loaded hydrogel. The density of capillaries in the scar tissue can be expressed through the expression of CD 34. CD 34 increases the mobility of vascular endothelial cells, which is useful for endothelium recovery and vascular reconstruction, and thus, its expression reflects the healing process [39]. The three-week period following surgical wound formation and complete re-epithelialization probably contributed to the lack of differences in scar vascularization between groups. Hydrogel treatment significantly inhibited the subsequent infiltration of Iba 1-positive cells (macrophages), compared to the control group, where their dominance was observed. Matrix metalloproteinases (MMPs) play a key role in wound re-epithelialization because they are involved in the regulation of angiogenesis, degradation, and deposition of the extracellular matrix [40]. Higher MMP-9 activity is associated with a decrease in fibroblast proliferation and activity, leading to impaired wound healing in diabetic patients. On the other hand, low levels of MMP-1 and MMP-9, which are found in the epithelialized wound, are associated with the end of the pathological condition [40]. Significantly reduced expression of MMP-9 in the group of animals treated with preparations based on immortelle has been observed, which confirmed the protective effects of the gel formulation. The results of the histopathological analysis correlate with the percentage of wound contraction and macroscopic characteristics and indicate that immortelle essential oil incorporated into hydrophilic polymers represents a promising strategy in the treatment of ulcers in patients with diabetes.

Oxidative stress is also associated with the disruption of the wound healing process in patients with diabetes. It occurs due to an imbalance in the generation of reactive oxygen species and the endogenous antioxidant defense mechanisms [41]. The parameters of the systemic redox status show a decrease in the level of pro-oxidants compared to the control group (Figure 7). Treatment with the immortelle essential oil-based hydrogel decreased the levels of pro-oxidants and increased the activity of antioxidant protection enzymes. This is in accordance with a study that shows that immortelle essential oil has antioxidative effects [42]. Consequently, it can be assumed that the reduction in oxidative stress is one of the mechanisms responsible for the acceleration of the wound healing process.

## 5. Conclusions

Hydrogel wound dressing loaded with immortelle essential oil possesses good viscoelastic properties and good spreadability and does not cause any signs of dermal irritation in vivo, thus appearing to be safe for cutaneous administration. Prominent and fast wound repair in rat excisional wound models confirm that immortelle essential oil-based hydrogel gel indeed has the desired wound healing properties and can be potentially used as a novel wound healing agent.

## Figures and Tables

**Table 1:** Swelling index of immortelle gel three days after preparation and 6 months of storage at 25 ± 2 °C.

	3rd Day			180 Days		
Sample/h	0	1	3	0	1	3
IG	87 ± 0.13	93 ± 0.11	130 ± 0.14	91 ± 0.21	96 ± 0.24	132 ± 0.15

The values are means of 3 replicates ± standard deviation. IG—immortelle essential oil-based gel.

**Table 2:** Surface of collagen.

Sample	Surface of Collagen (µm^2^)
C	61,837
SSD	91,236
GB	31,932
IG	242,424

C—control group; SSD—silver sulfadiazine; GB—gel base; IG—immortelle gel.

## Data Availability

Data are contained within the article.
