# Correction: Andjic et al. Immortelle Essential-Oil-Enriched Hydrogel for Diabetic Wound Repair: Development, Characterization, and In Vivo Efficacy Assessment. *Pharmaceutics* 2024, *16*, 1309

**DOI:** 10.3390/pharmaceutics17081072

**Published:** 2025-08-19

**Authors:** Marijana Andjic, Jovana Bradic, Aleksandar Kocovic, Marko Simic, Veljko Krstonosic, Ivan Capo, Vladimir Jakovljevic, Nevena Lazarevic

**Affiliations:** 1 Department of Pharmacy, Faculty of Medical Sciences, University of Kragujevac, 34000 Kragujevac, Serbia; andjicmarijana10@gmail.com (M.A.); simic.marko.kg@gmail.com (M.S.); nevenasdraginic@gmail.com (N.L.); 2 Center of Excellence for Redox Balance Research in Cardiovascular and Metabolic Disorders, 34000 Kragujevac, Serbia; drvladakgbg@yahoo.com; 3 Department of Pharmacy, Faculty of Medicine, University of Novi Sad, 21000 Novi Sad, Serbia; veljkokrst@yahoo.co.uk; 4 Department of Histology and Embryology, Faculty of Medicine, University of Novi Sad, 21000 Novi Sad, Serbia; ivan.capo@mf.uns.ac.rs; 5 Center for Medical and Pharmaceutical Investigations and Quality Control, University of Novi Sad, 21000 Novi Sad, Serbia; 6 Department of Physiology, Faculty of Medical Sciences, University of Kragujevac, 34000 Kragujevac, Serbia; 7 Department of Human Pathology, 1st Moscow State Medical, University IM Sechenov, 119991 Moscow, Russia

## Missing Acknowledgment

In the original publication [1], the Acknowledgments section should contain the following statement: This research was supported by the Science Fund of the Republic of Serbia, #GRANT No 11067, *Unleashing Nature*’*s Potential*: *Using Grape Skin Extract and Sustainable Materials for Advanced Chronic Wound Therapy*-GraSP_MAT.

The authors state that the scientific conclusions are unaffected. This correction was approved by the Academic Editor. The original publication has also been updated.
